# Cell Transfection with a β-Cyclodextrin-PEI-Propane-1,2,3-Triol Nanopolymer

**DOI:** 10.1371/journal.pone.0100258

**Published:** 2014-06-23

**Authors:** Wing-Fu Lai, Han-Sung Jung

**Affiliations:** 1 Division in Anatomy and Developmental Biology, Department of Oral Biology, Oral Science Research Center, BK21 PLUS Project, Yonsei University College of Dentistry, Seoul, Korea; 2 Oral Biosciences, Faculty of Dentistry, The University of Hong Kong, Hong Kong SAR; Osaka University Graduate School of Medicine, Japan

## Abstract

Successful gene therapy necessitates safe and efficient gene transfer. This article describes the use of a cationic polymer, which was synthesized by cross-linking low molecular weight branched poly(ethylenimine) (PEI) with both β-cyclodextrin and propane-1,2,3-triol, for efficient and safe non-viral gene delivery. Experimentation demonstrated that the polymer had a pH buffering capacity and DNA condensing ability comparable to those of PEI 25 kDa. In B16-F0 cells, the polymer increased the transfection efficiency of naked DNA by 700-fold and yielded better transfection efficiencies than Fugene HD (threefold higher) and PEI 25 kDa (fivefold higher). The high transfection efficiency of the polymer was not affected by the presence of serum during transfection. In addition to B16-F0 cells, the polymer enabled efficient transfection of HepG2 and U87 cells with low cytotoxicity. Our results indicated that our polymer is a safe and efficient transfection reagent that warrants further development for *in vitro*, *in vivo* and clinical applications.

## Introduction

Gene delivery by viral and non-viral vectors is an important area of research due to its potential applications in gene therapy. Though viral vectors generally have higher transfection efficiency, their immunogenicity and safety risks make them an unfavorable choice for clinical applications. Over the years, numerous non-viral gene delivery systems have been reported [1]–[4]. Among these systems, poly(ethylenimine) (PEI) has gained special attention because of its high transfection efficiency [2]. PEI is a polyamine which possesses a high proton buffering capacity over a wide pH range [5]. Compared with its linear counterpart, branched PEI (bPEI) has higher primary amine content. As primary amines generally are more reactive than secondary amines, bPEI is easier to modify [6] and has thus been favored over linear PEI in gene delivery research [2]. In fact, as early as the 1990's, bPEI 25 kDa had already been exploited as a gene carrier [7]. The polymer and its derivatives also emerged as favorites for delivering various nucleic materials (such as ribozymes [8], plasmids [9] and oligonucleotides [10]) for animal studies. Though bPEI 25 kDa exhibited high transfection efficiency, its toxicity was also high. The latter observation was supported by the 8.6% mortality rate in mice 24 hours after nasal instillation of bPEI 25 kDa [11]. Such high toxicity has impeded the practical application of bPEI 25 kDa.

To reduce toxicity, low molecular weight (LMW) bPEI has been exploited as an alternative gene carrier [12]. Though LMW bPEI exhibited lower toxicity, its transfection efficiency was measurably inferior to that obtained with high molecular weight bPEI [13]. This result had been demonstrated by an earlier study [13], which found that in human endothelial cell-derived EA.hy 926 cells, PEI with a molecular weight of 70 kDa yielded an average transfection efficiency of approximately 20–30%, whereas PEI with a molecular weight less than or equal to 1.8 kDa produced little or no transfection. In this study, we linked LMW bPEI first with propane-1,2,3-triol, which functions as a biodegradable linker generating a biodegradable, higher molecular weight construct of PEI. The product was further cross-linked with β-cyclodextrin (β-CD) to form a nanopolymer with a star-shaped architecture. That nanopolymer was designated as β-CD-bPEI-propane-1,2,3-triol (BPEA). The transfection efficiency of BPEA was not only comparable, or even superior, to that obtained using bPEI 25 kDa or Fugene HD in various tumor cell lines, but also produced little or no toxicity. Our results indicate that BPEA is a novel nanopolymer that warrants further development as a gene carrier for both *in vitro* and *in vivo* transfection.

## Materials and Methods

### Materials

#### Chemicals

bPEI (Mw = 1.2 and 25 kDa), β-cyclodextrin, dimethyl sulfoxide (DMSO), triethylamine (Et_3_N), propane-1,2,3-triol, and 1,1′-carbonyldiimidazole (CDI) were purchased from Sigma–Aldrich (St. Louis, MO, USA). bPEI 25 kDa was purified by dialysis against triple distilled water, then lyophilized before use.

#### Plasmids

The plasmid pEGFP-N1 and the pGL3 Luciferase reporter vector were purchased from BD Biosciences (San Jose, CA) and Promega Corporation (Madison, WI), respectively. They were transformed into competent DH5α cells, and plated onto LB plates supplemented with 100 µg/mL ampicillin. Plasmids were then isolated using the PureLink Hipure Plasmid Maxiprep kit (Invitrogen) according to the manufacturer's guidelines. The quality and quantity of the purified plasmids were analyzed by measuring their optical densities at 260 and 280 nm.

#### Cell culture

Dulbecco's Modified Eagle's Medium (DMEM), Minimum Essential Medium (MEM), trypsin–EDTA (0.25% trypsin–EDTA), penicillin–streptomycin (10,000 units penicillin and 10 mg streptomycin/ml) and fetal bovine serum (FBS) were obtained from Invitrogen. Human glioblastoma U87 cells, murine melanoma B16 cells and human hepatocellular liver carcinoma HepG2 cells were purchased from ATCC (American Type Culture Collection).

#### Animals

Normal female C57/BL6 mice were purchased from Daehan Laboratory Animal Research Center (Korea). All animal works were conducted in accordance with the protocol approved by the Animal Care and Use Committee of Yonsei Medical Center, Seoul, Korea.

### Methods

#### Synthesis of BPEA

Propane-1,2,3-triol was dissolved in degassed DMSO at a concentration of 0.17 g/ml and mixed with a DMSO solution (0.3 g/ml) of CDI. Et_3_N was added to the reaction mixture at a concentration of 33 µl/ml. The reaction was conducted in the dark for 3 hours under an inert nitrogen atmosphere. The reaction mixture was then diluted with 5 ml of degassed DMSO, followed by an addition of a DMSO solution (0.88 g/ml) of bPEI 1.2 kDa. The reaction was carried out for 16 hours in the dark at 37°C under an inert nitrogen atmosphere. The crude reaction mixture was dialyzed against triple distilled water at 4°C for 3 days with a molecular weight cutoff of 2 kDa. The solution was then filtered using syringe filters and lyophilized for 2 days to obtain bPEI-propane-1,2,3-triol (PEA).

To synthesize BPEA, β-CD and CDI were first mixed at a 1∶1 ratio. The mixture was then dissolved in degassed DMSO at a concentration of 0.2 g/ml. Et_3_N was added to the reaction mixture at a concentration of 33 µl/ml. The reaction was carried out for 3 hours in the dark under an inert nitrogen atmosphere. Afterwards, 14 ml of a DMSO solution of PEA were added dropwise into the reaction mixture. The reaction was carried out for 16 hours and was performed in the dark at 37°C under an inert nitrogen atmosphere. The crude reaction mixture was dialyzed against triple distilled water at 4°C for 3 days with a molecular weight cutoff of 12.4 kDa. The solution was then filtered by syringe filters. BPEA was obtained after lyophilization for 2 days.

#### Proton nuclear magnetic resonance (^1^H-NMR)

20 mg of BPEA, PEA or PEI was solubilized in 0.7 ml of deuterium oxide (D_2_O). ^1^H-NMR spectra was recorded on an NMR spectrometer (500 MHz; Bruker Corporation, Germany).

#### Fourier-transform infrared (FT-IR) spectroscopy

FT-IR spectroscopy was performed using an FT-IR spectrometer (Spectrum 2000, PERKIN ELMER) at ambient conditions. The potassium bromide (KBr) disk technique was used for analysis. Spectra were obtained at a resolution of 2 cm^−1^, and reported as an average of 16 scans.

Matrix-assisted laser desorption-ionization time-of-flight (MALDI-TOF) mass spectrometry. The matrix solution was prepared by dissolving 10 mg of the matrix (2,5-dihydroxybenzoic acid/fucose  = 1∶1) in 1 ml of 1∶1 water/acetonitrile mixture. This solution was then mixed with an aqueous solution of a sample (0.5 mg/ml) in a 1∶1 ratio before analysis. MALDI-TOF mass spectrometry was performed using a Voyager Biospectrometry Workstation mass spectrometer (Applied Biosystems, Foster City, CA, USA), equipped with a pulsed N_2_ laser (λ = 337 nm, 2 ns pulse width) and operated in the linear delayed-extraction mode. The acceleration voltage adopted was 22 kV.

#### Polyplex formation and electron microscopy

BPEA/DNA polyplexes were prepared by adding polymer solution (in triple distilled water) to an equivalent volume of plasmid solution (in Tris/EDTA buffer) at an appropriate BPEA/DNA mass-to-mass ratio. The mixture was vortexed gently for 10 seconds, followed by incubation at room temperature for 30 minutes.

The surface morphologies of BPEA/DNA polyplexes were characterized using both scanning electron microscopy (SEM) and transmission electron microscopy (TEM). In brief, ten microliters of a polyplex solution were dried onto a Joel specimen copper support. The polyplexes were imaged on a LaB6 Joel CX120 II with ASID scanning attachment at 20 kV. Apart from this, a drop of the polyplex solution containing 0.1 wt% phosphotungstic acid (PTA) was placed on a 200-mesh carbon-coated copper grid. After evaporation of the solvent at ambient conditions for 5 min, images were taken using a transmission electron microscope (JEM-2010 TEM) operating at an acceleration voltage of 100 kV.

#### Particle size and zeta potential measurement

The size and surface charge of the formed polyplexes were examined by laser-light scattering (Zetasizer 3000, Malvern Instruments, UK). Data were reported as an average of 10 measurements.

#### Gel retardation assay

The pDNA condensing ability of the polymer of interest was studied by the gel retardation assay. After the formation of polyplexes, the polyplexes were mixed with the loading buffer, stained with Loading STAR (DYNE Bio, Seongnam, Korea), and electrophoresed in a 1% agarose gel at 80 V for one hour.

#### pH buffering capacity assay

The pH buffering capacities of BPEA and bPEI 25 kDa were evaluated as previously described [14].

#### In vitro transfection efficiency assay

Different cancer cell lines (U87, B16 and HepG2) were seeded separately in 24-well plates at a density of 1×10^5^ cells per well, and incubated at 37°C for 24 hours. During transfection, the growth medium was replaced with 300 µl of fresh medium with or without 10% FBS. Preparation of the polyplexes of BPEA/pGL3 or BPEA/pEGFP-N1 was performed by mixing 2 µg of the plasmid with an appropriate quantity of BPEA at appropriate BPEA/DNA mass-to-mass ratios. The mixture was vortexed gently for 10 seconds, followed by incubation at room temperature for 30 minutes. Triple distilled water was then added volumetrically to the mixture to reach a final volume of 100 µl. The mixture was added to each well. After 5-hour incubation at 37°C, the transfection medium was replaced with fresh growth medium. The cells were incubated at 37°C for an additional 24 hours.

During experiments, Fugene HD and bPEI 25 kDa were used as positive controls. The transfection efficiency of BPEA was determined by the luciferase activity assay as previously described [14]. Microscopic evaluation of EGFP expression in transfected cells was also performed according to a previously published method [15]. To perform flow cytometry analysis, the transfected cells were trypsinized and re-suspended in PBS. Fluorescence-activated cell sorting (FACS) of EGFP-positive cells was conducted using a flow cytometer (Epics XL; Coulter, Hialeah, FL). Non-transfected cells were adopted to set the background.

#### Cytotoxicity assay

Different cancer cell lines (U87, B16 and HepG2) were seeded separately in 96-well plates at an initial density of 5,000 cells per well, and incubated at 37°C for 24 hours. The growth medium was replaced with 100 µl of fresh cell culture medium with or without 10% FBS. 10 µl of the polymer solution was added to each well. After 5-hour incubation at 37°C, the polymer containing medium was replaced with 100 µl of fresh growth medium. The cytotoxicity of the polymer was evaluated using the Cell Count Kit-8 (CCK-8) according to the manufacturer's guidelines. Cell viability (%) in each well was determined by dividing the absorbance value (A_450_) of the test well by the A_450_ value of the control well, followed by a multiplication of the quotient by 100.

#### Blood collection and hemolysis assay

Blood from anesthetized mice via cardiac puncture was collected into a heparin-containing tube, and centrifuged for 10 minutes at 2,000 g at 4°C. The collected erythrocytes were washed with PBS (pH = 7.4) until the supernatant was colorless. Polyplexes of BPEA and PEI 25 kDa, at various polymer/DNA ratios, were freshly prepared in PBS before use. The collected erythrocytes were added to reach a final concentration of 8% (v/v). After incubation at 37°C for one hour, the mixture was centrifuged at 2,000 g for 15 minutes. The absorbance of the supernatant was recorded at 414 nm. Experiments were performed in triplicate. The extent of hemolysis in PBS and 0.1% Triton X-100 was defined as 0% and 100%, respectively.

## Results

### Fabrication and characterization of BPEA

#### Synthesis of PEA and BPEA

**Figure 1:**
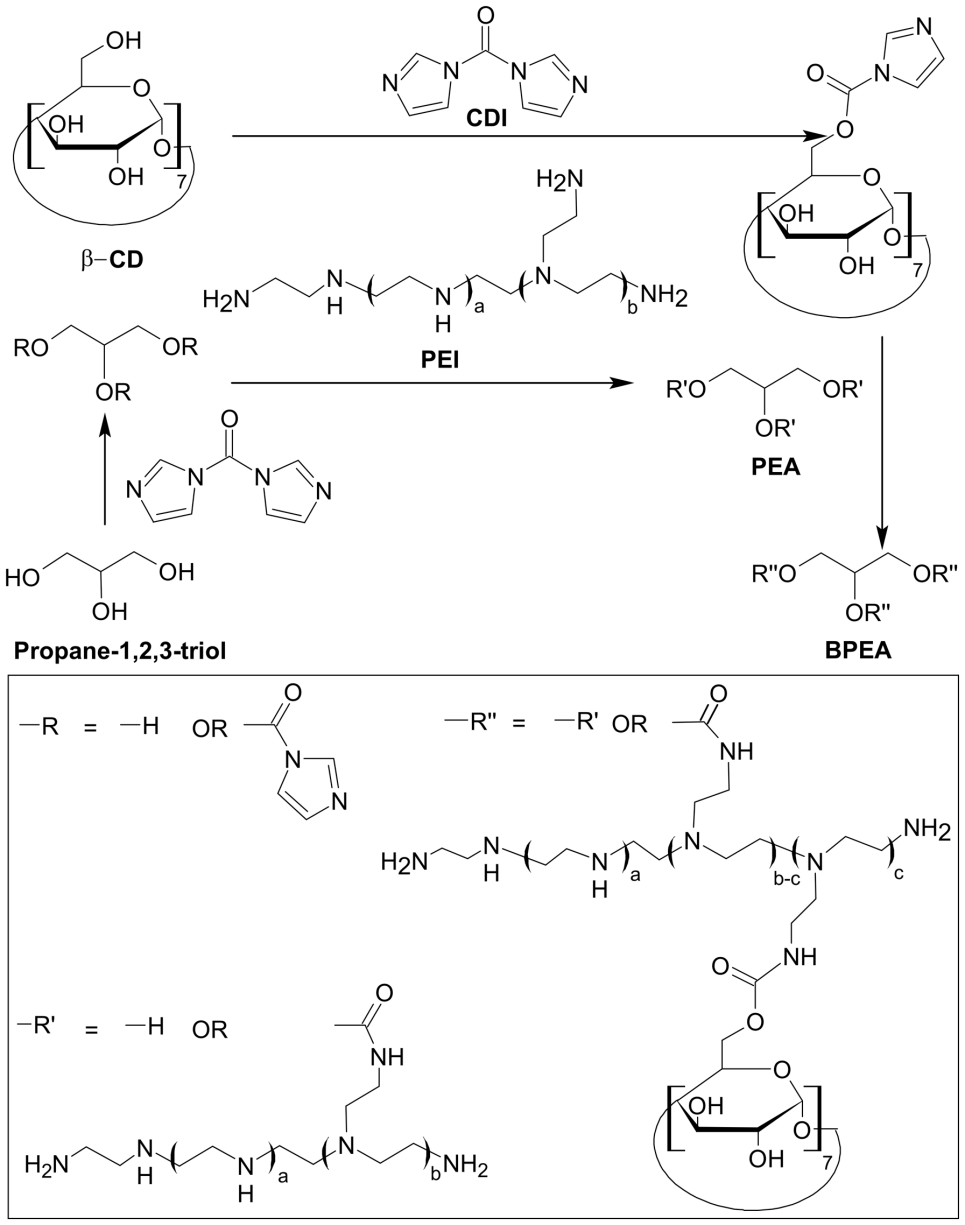
Synthesis and putative structure of BPEA.

The procedure for synthesizing BPEA is shown in Fig. 1. The synthetic procedure consisted of two stages. In the first stage, the hydroxyl groups of propane-1,2,3-triol were activated by CDI. Active imidazolyl carbamate intermediates were formed and subsequently attacked by the primary amine groups of PEI to generate PEA. In the second stage, CDI activated the hydroxyl groups of β-CyD, which in turn reacted with the primary amine groups of PEA to generate BPEA. Since the molecular weight cutoff (12.4 kDa) of the dialysis membrane used to isolate BPEA was much higher than the molecular weight of all reactants used throughout the experiments, the dialysis process affords the product in high purity.

#### NMR and FT-IR studies

**Figure 2:**
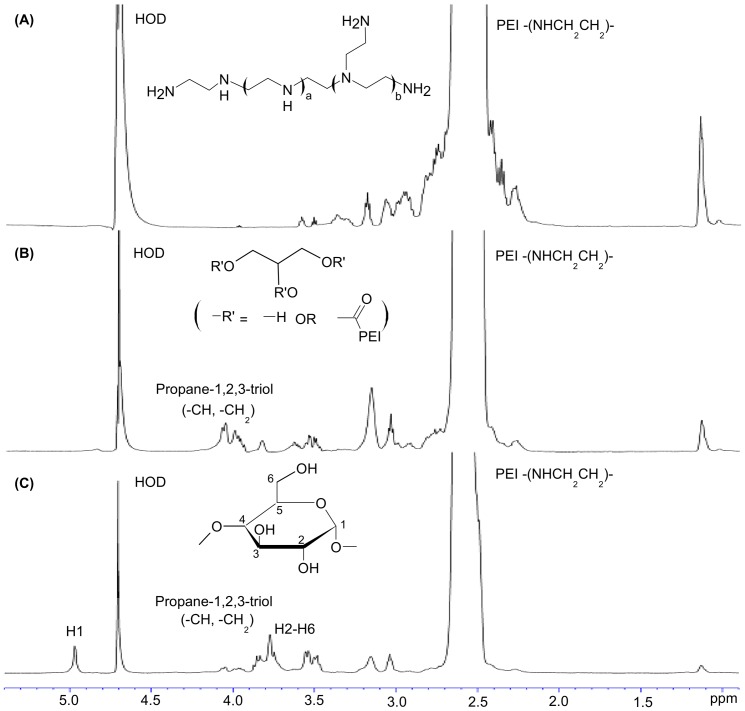
^1^H-NMR spectra of (A) bPEI 12 kDa, (B) PEA and (C) BPEA.

According to Fig. 2, representative signals of PEI (-CH_2_CH_2_N- protons) in D_2_O resonate between 2.5 and 3.2 ppm (Fig. 2A). The presence of peaks, which resonate between 3.7 ppm and 4.1 ppm, from propane-1,2,3-triol (CH, CH_2_) demonstrated the successful generation of PEA (Fig. 2B). The NMR spectrum of BPEA is shown in Fig. 2C. The presence of the representative signal at 4.95 ppm (C-1 hydrogen in β-CyD) in the spectrum of BPEA suggested the successful incorporation of β-CyD into PEA. The molar ratio of PEI to β-CyD to propane-1,2,3-triol in BPEA was calculated based on the proton integral values of the ^1^H NMR spectrum: 2.3–2.8 ppm (CH_2_ of PEI), 4.95 ppm (C-1 hydrogen in β-CyD) and 3.7–4.1 ppm (CH and CH_2_ from propane-1,2,3-triol) and was approximated to be 5∶1∶6.

**Figure 3:**
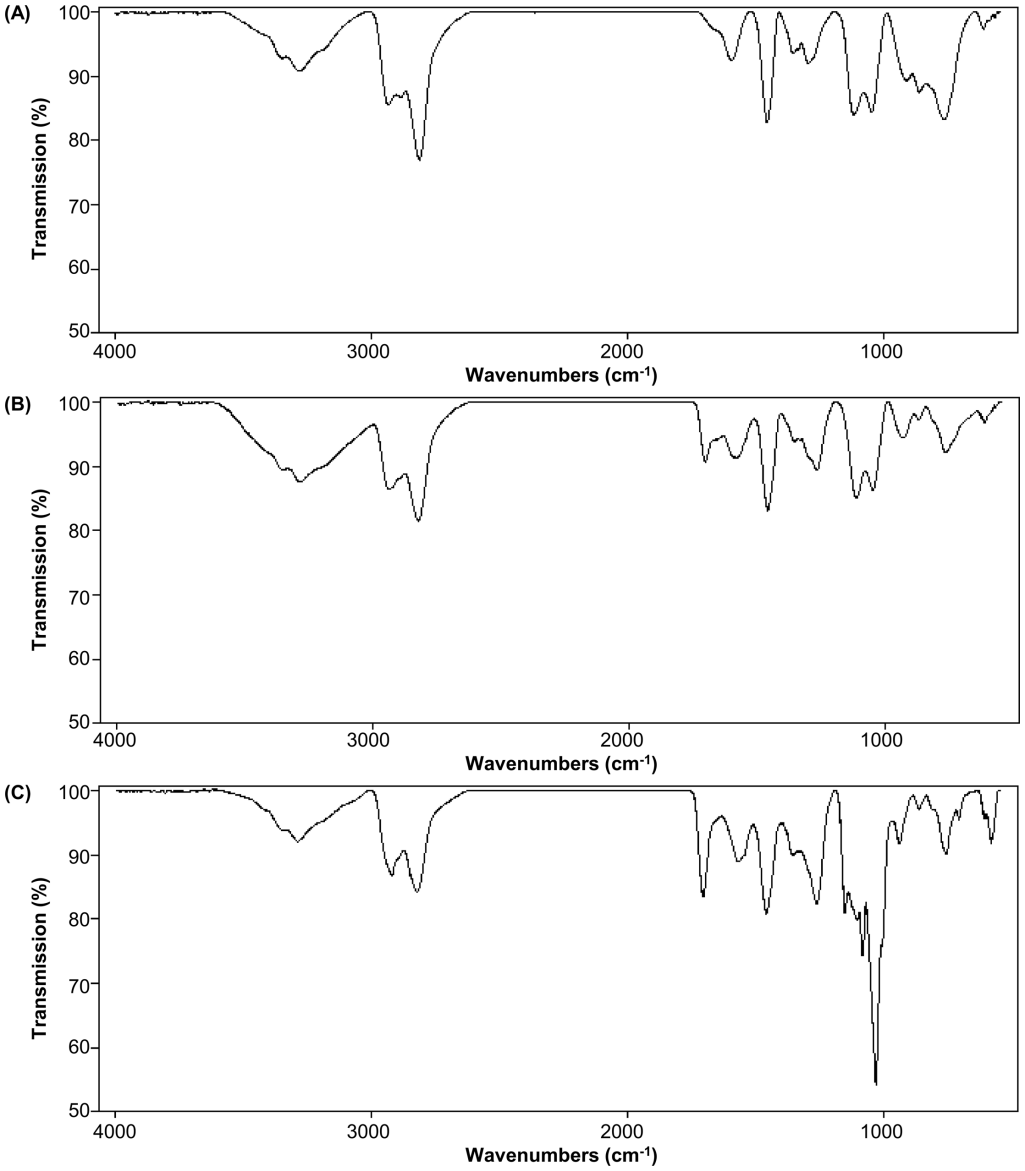
FT-IR spectra of (A) bPEI 12 kDa, (B) PEA and (C) BPEA.

The successful generation of PEA and BPEA was further confirmed using FT-IR. In the spectrum of PEI (Fig. 3A), stretching vibration peaks of proton assigned to methyl and methylene could be found at 2920 cm^−1^ and 2822 cm^−1^. After cross-linking of PEI with propane-1,2,3-triol and due to the hydrogen bonding between N-H of PEI and C = O of propane-1,2,3-triol, an ester signal shifted to 1703 cm^−1^ in the spectrum of PEA (Fig. 3B). Finally, apart from the wide peak caused by the OH group at around 3200–3400 cm^−1^, O-C stretching vibration peaks of β-CD appeared at 1032, 1083 and1153 cm^−1^ in the spectrum of BPEA (Fig. 3C), suggesting successful incorporation of β-CD into PEA.

#### MALDI-TOF

**Figure 4:**
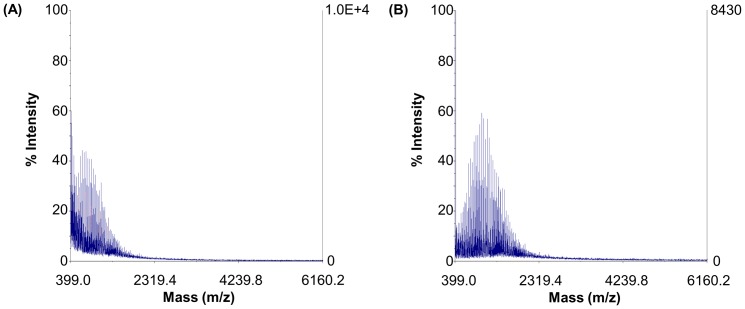
MALDI-TOF mass spectra of (A) PEA and (B) BPEA.

BPEA and PEA were further characterized using MALDI-TOF. Intensity signals for PEA were found extensively in a range between 499.0 and 8253.6 (m/z), with the highest peaks being found at around 706.9 (m/z) (Fig. 4A). On the other hand, most of the intensity signals in the spectrum of BPEA appeared from 513.6 to 19873.9 (m/z), with the highest peaks being found at 1137.4 (m/z) (Fig. 4B). Due to the high polydispersity of our polymers and the resulting mass discrimination against high-mass oligomers in the MALDI-TOF spectra [16], the molecular weight distribution (MWD) of PEA and BPEA can hardly be accurately determined using the results. However, compared with the position of the peaks in the spectrum of PEA, peaks in the spectrum of BPEA have been shifted towards the higher mass range. Such a shift indicated that the molecular mass of BPEA is higher than PEA, suggesting successful cross-linking of PEA by β-CyD.

#### Physical characterization of BPEA

**Figure 5:**
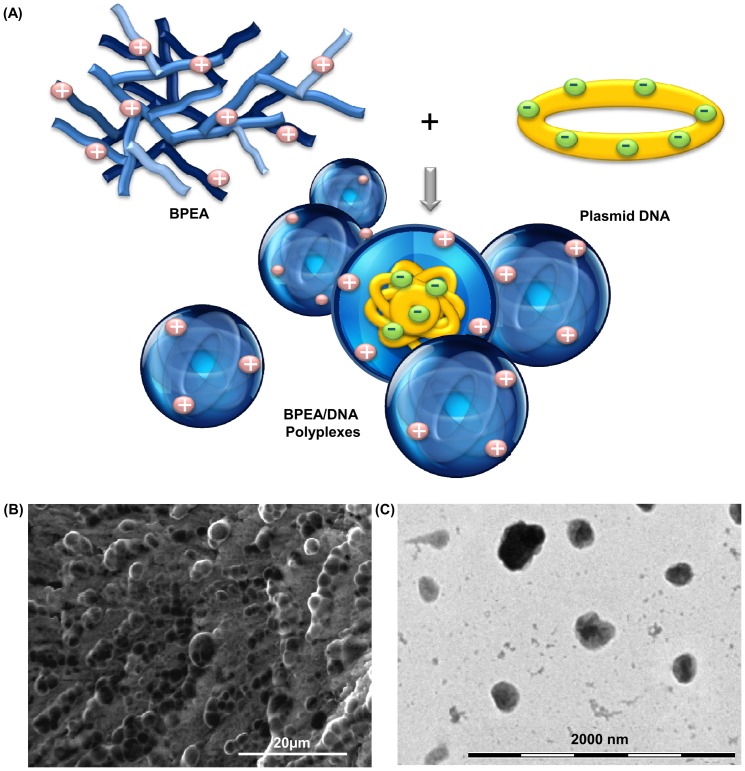
Polyplex formation of BPEA with DNA. (A) Schematic representation of the formation of BPEA/DNA polyplexes via electrostatic interactions between BPEA and plasmid DNA. (B) A representative scanning electron micrograph and (C) transmission electron micrograph of the polyplexes formed by BPEA at the polymer/DNA ratio of 60.

**Figure 6:**
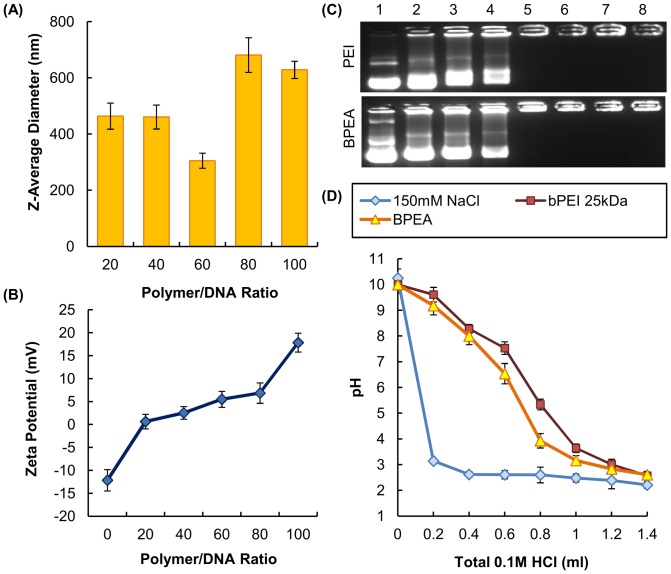
Physical characterization of BPEA and its polyplexes. (A) The diameters of the polyplexes measured by dynamic light scattering. (B) The zeta potential of polyplexes formed by BPEA at different polymer/DNA ratios. (C) Gel retardation assay of PEI 25 kDa and BPEA. The polymer/DNA ratios used in lanes 1, 2, 3, 4, 5, 6, 7 and 8 were 0, 0.03, 0.06, 0.125, 0.25, 0.5, 1 and 2, respectively. (D) The acid-base titration profiles of PEI 25 kDa and BPEA. The solvent, 150 mM NaCl, was used as a control.

A schematic representation of the polyplex formation process between BPEA and DNA plasmids was illustrated in Fig. 5A. It is expected that polyplexes were formed via mainly electrostatic interactions. As shown by SEM, the morphology of BPEA/DNA polyplexes was as compact spheres (Fig. 5B). The particle size of BPEA polyplexes formed at the optimal polymer/DNA ratio (60/1) was estimated by TEM to be 283.3±40.8 nm (Fig. 5C), with a unimodal size distribution from 240 to 320 nm. This was consistent with the particle size determined by dynamic light scattering (DLS), which found that at a polymer/DNA ratio of 60/1, BPEA/DNA polyplexes had the smallest particle size and had a Z-average diameter of approximately 270–330 nm (Fig. 6A).

In addition, the zeta potential of BPEA polyplexes was positive at all N/P ratios tested (Fig. 6B). Results of the gel retardation assay showed that BPEA can completely retard DNA at a polymer/DNA ratio of 0.25/1, and such a DNA condensing ability was comparable to that of PEI 25 kDa (Fig. 6C). As determined by acid-base titration, the pH buffering capacity of BPEA was comparable to that of bPEA 25 kDa (Fig. 6D).

### In vitro transfection

**Figure 7:**
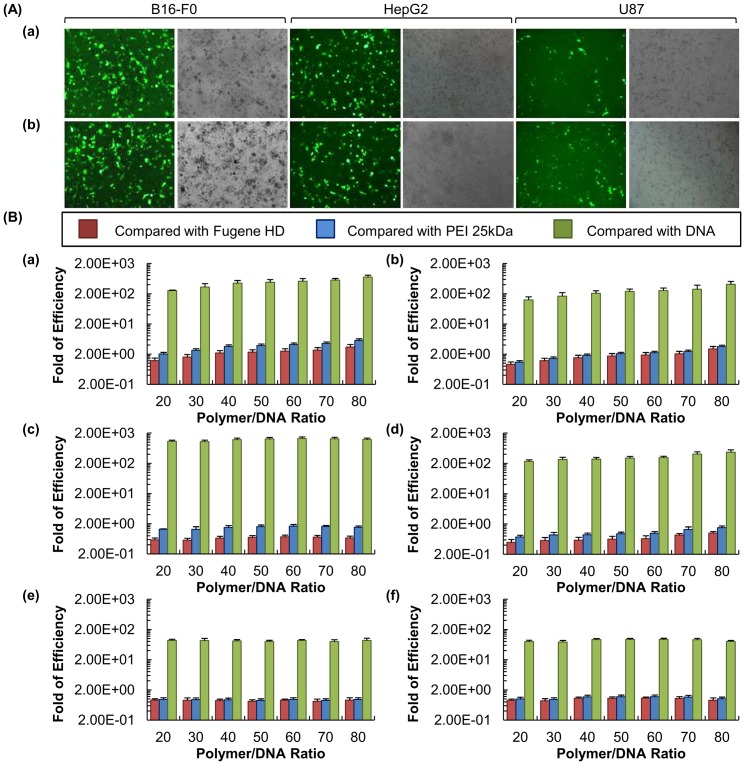
Gene transfection efficiency of BPEA/DNA polyplexes. (A) Fluorescence and differential interference contrast images of B16-F0, HepG2 and U87 cells transfected with BPEA in the (a) absence and (b) presence of 10% FBS. (B) The fold of transfection efficiency obtained using BPEA when the percentage of EGFP-positive (a and b) B16-F0, (c and d) HepG2 and (e and f) U87 cells transfected with BPEA, at different polymer/DNA ratios, was compared with those achieved using Fugene HD, PEI 25 kDa and DNA alone, both in the (a, c and e) absence and (b, d and f) presence of 10% FBS.

Microscopic evaluation of EGFP expression.The fluorescence and differential interference contrast images of B16-F0 cells transfected with BPEA at different polymer/DNA ratios are shown in Fig. 7A. The EGFP fluorescence yielded by BPEA-mediated transfection was quantitatively assessed by flow cytometry (Fig. 7B). The highest transfection efficiency obtained by BPEA was 3-fold and 5-fold higher than that achieved by Fugene HD and PEI 25 kDa, respectively (Fig. 7Ba). Compared with using DNA alone, BPEA can increase the transfection efficiency up to 700 fold (Fig. 7Ba). Such a high transfection efficiency was maintained even in the presence of 10% FBS (Fig. 7Bb). Apart from B16-F0 cells, the high transfection efficiency of BPEA was confirmed in HepG2 and U87 cells. The flow cytometry results showed that the highest transfection efficiency attained by BPEA in these two cell lines was comparable to that of Fugene HD and PEI 25 kDa, being far superior to that of the naked plasmid (Fig. 7Bc–f).

#### Luciferase activity assay

**Figure 8:**
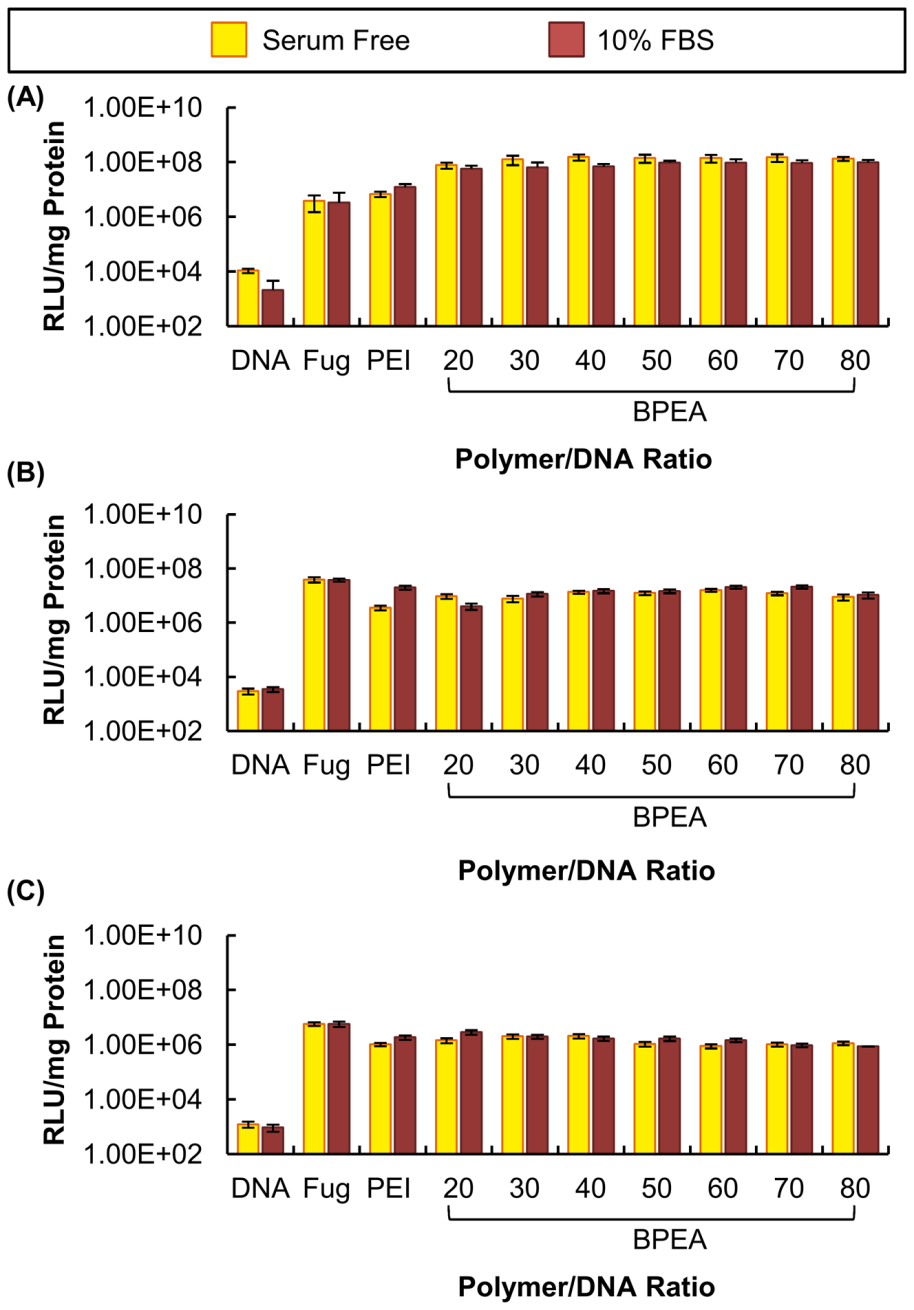
Comparison of luciferase expression mediated by BPEA (with different polymer/DNA ratios), PEI 25 kDa, Fugene HD and DNA alone. The experiment was performed in (A) B16-F0, (B) HepG2 and (C) U87 cells both in the absence and presence of 10% FBS.

In addition to the EGFP transfection efficiency assay, the transfection efficiency of BPEA was assessed using a luciferase activity assay (Fig. 8). The results showed that B16-F0 cells transfected with BPEA had 1–2 orders of magnitude higher luciferase activity than those transfected with Fugene HD and PEI 25 kDa and 4–5 orders of magnitude higher than those transfected with DNA alone. In HepG2 and U87 cells, the luciferase activity of the cells transfected with BPEA was also close to that of the cells transfected with Fugene HD and PEI 25 kDa. The high transfection efficiency of BPEA was not influenced by the presence of serum during transfection.

#### Cytotoxicity and hemolysis

**Figure 9:**
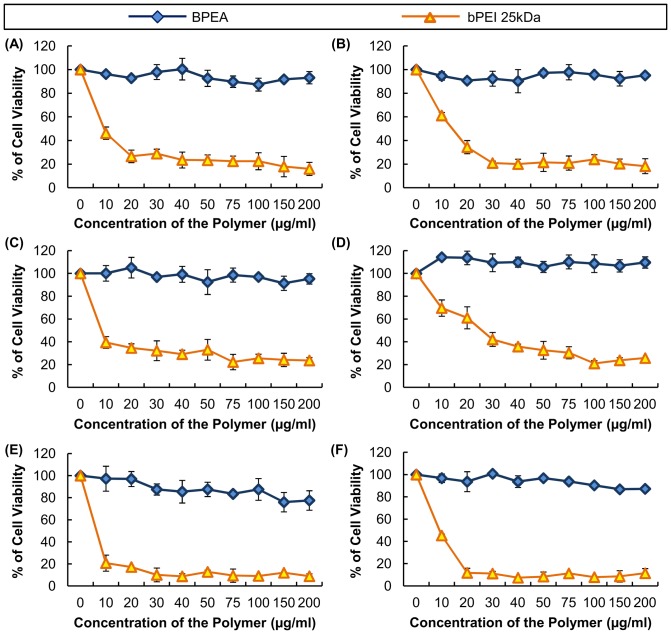
The cytotoxicity of BPEA. The experiment was performed in (A and B) B16-F0, (C and D) HepG2 and (E and F) U87 cells both in the (A, C and E) absence and (B, D and F) presence of 10% FBS. PEI 25 kDa was used as a positive control to compare the relative cytotoxicity of BPEA.

**Figure 10:**
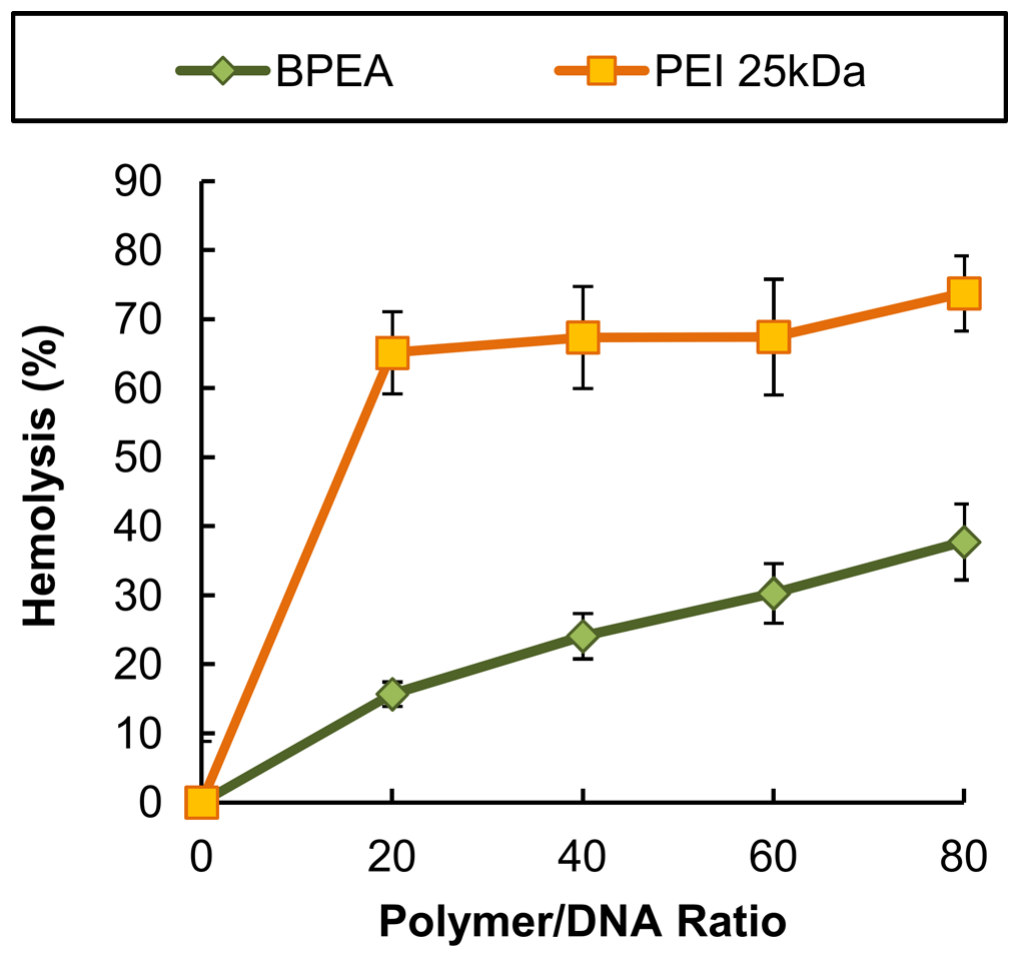
The hemolytic effects of PEI 25/DNA and BPEA/DNA polyplexes on mouse erythrocytes.

The cytotoxicity of BPEA in B16-F0, HepG2 and U87 cells in the absence and presence of 10% FBS is shown in Fig. 9. The results suggested that, up to a concentration of 200 µg/ml, BPEA was basically non-toxic to all the cell lines tested. Comparatively, cells treated with PEI 25 kDa displayed a drastic drop in their viability with increasing concentrations of the polymer. The results of our hemolysis assay (Fig. 10) also showed that PEI 25 kDa induced approximately 60–80% hemolysis, which was approximately 2–4 times higher than the extent of hemoglobin release induced by BPEA.

## Discussion

Over the years, PEI has been widely exploited as a candidate vector for gene delivery due to its high transfection efficiency; however, its clinical practicality has been hampered by its high toxicity during transfection. An earlier study has compared the transfection efficiency of PEIs with different molecular weights (600, 1200, 1800, 10,000, and 70,000 Da) in endothelial cells [13]. The study found that the transfection efficiency of PEI was positively correlated with the molecular weight of PEI, and PEIs with molecular weights below 1,800 Da essentially failed to transfect cells. Given this fact, in this study we have cross-linked LMW PEI with propane-1,2,3-triol via CDI-mediated coupling reactions (Fig.1) to generate a biodegradable, higher molecular weight construct of PEI, namely PEA. The rise in the molecular weight after cross-linking is expected to augment the transfection efficiency of the originally uncross-linked LMW PEI. But due to the high biodegradability of propane-1,2,3-triol [17], the impact of the increase in the molecular weight on cytotoxicity is minimal.

To further augment the efficiency in gene delivery, PEA was cross-linked with β-cyclodextrin (β-CD) to generate a nanopolymer with a star-shaped architecture. The viability of employing cyclodextrins as linking agents to improve the performance of gene carriers has been suggested by an earlier study, which linked PEI with β-cyclodextrin and observed that the resulting product induced an approximately four-fold increase in luciferase expression compared with that induced by unmodified PEI [18]. More recently, a series of star polymers have been reported by Yang et al., who conjugated multiple oligoethylenimine arms onto an α-cyclodextrin core [19]. The star polymers showed high transfection efficiency in HEK293 and Cos7 cells, and had cytotoxicity much lower than that of bPEI 25 kDa. Since current advances in applications of cyclodextrins to non-viral gene delivery have been reviewed by a recent article [4], readers are referred to that article for details [4]. In general, cyclodextrins have high potential in gene transfer because of their binding affinity to nucleic acids [20], [21], their ability to attenuate the cytotoxicity of other gene vectors [22], and their absorption-enhancing property in therapeutics delivery [23], [24]. Given this fact, the performance of PEA in gene delivery is expected to be augmented upon cross-linking with β-CD.

The structure of BPEA was characterized by NMR. The presence of peaks resonating between 3.7 ppm and 4.1 ppm from propane-1,2,3-triol (CH, CH_2_), 2.5 and 3.2 ppm from PEI (-CH_2_CH_2_N- protons), and the representative signal at 4.95 ppm from β-CD (C-1 hydrogen) demonstrated successful generation of BPEA (Fig. 2). The success of BPEA formation was further confirmed by the presence of the following three groups of signals in the FT-IR spectrum of BPEA (Fig. 3): (a) an ester signal at around 1700 cm-1, (b) O-C stretching vibration peaks of β-CD at around 1030–1150 cm^−1^, and (c) stretching vibration peaks of methyl and methyne protons in PEI at around 2820–2920 cm^−1^. Moreover, as shown by the MALDI-TOF mass spectra of PEA and BPEA, peaks in the spectrum of BPEA have been shifted towards the higher mass range as compared with those in the spectrum of PEA (Fig. 4). Such a shift indicated that the molecular mass of BPEA is higher than PEA, suggesting successful cross-linking of PEA by β-CyD.

The cytotoxicity of BPEA was determined by our cytotoxicity test, which revealed that in all of the cell lines tested (i.e., U87, HepG2 and B16-F0), BPEA had low cytotoxicity over a wide range of concentrations (Fig. 9). The low cytotoxicity of BPEA may be attributed to the low charge density of its polyplexes. As suggested by a previous study, the cytotoxicity of PEI 25 kDa was partially due to the membrane-disruptive property of the dense cationic polymers [25]. This effect was supported by the observation that at an N/P ratio of 10 or higher, the zeta potential of polyplexes formed by PEI 25 kDa was usually higher than +30 mV [26]. However, the zeta potential of polyplexes formed by BPEA was less than +20 mV at all ratios tested (Fig. 6). Such low charge density of the BPEA polyplexes may explain the decreased disruption of the cell membrane, and hence the lower cytotoxicity, when compared with PEI 25 kDa. The lower membrane-disrupting activity of BPEA was confirmed by the hemolysis assay (Fig. 10), which showed that BPEA induces a much lower degree of hemoglobin release from mouse erythrocytes than does PEI 25 kDa.

With regards to polymeric gene delivery, one of the hurdles to overcome is the cell membrane, which acts as a selective permeable barrier to govern the transport of substances into and out of the cell. Accordingly, the particle size of the polyplex strongly influences the efficiency of its cellular uptake and the resulting transfection efficiency. Our results showed that at a polymer/DNA ratio of 60, the particle size of the polyplexes formed by BPEA was approximately 250–350 nm (Fig. 6), which is in the range for effective endocytosis. In addition, complexation of DNA with the cationic polymer (Fig. 5) will neutralize the negative charge of the DNA molecule. As the polymer/DNA ratio increases, the surface charge of the polyplex will increase gradually [27], [28]. A positive surface charge facilitates binding of the polyplex to the anionic cell surfaces for cellular internalization [29]. As the zeta potential of BPEA polyplexes was positive at all polymer/DNA ratios tested, this characteristic may also partially facilitate cellular internalization of the polyplexes. Once the polyplexes have been internalized, they must then undergo endo-lysosomal escape to avoid possible lysosomal degradation. Our results showed that the pH buffering capacity of BPEA was comparable to that of PEI 25 kDa, which has a high proton buffering capacity over a broad range of pH values (Fig. 6). As high buffering capacity has been associated with great proton sponge effects, which can result in osmotic swelling and rupture of endosomes, this suggests that polyplexes formed by BPEA may efficiently disrupt the vesicle membrane to release the polyplexes into cytoplasm.

The high transfection efficiency of BPEA was confirmed by the EGFP transfection efficiency assay in B16-F0, HepG2 and U87 cells. As shown in Fig. 7A, the cells transfected with BPEA had extensive EGFP expression. In order for a gene carrier to be applicable *in vivo* or clinically, it must be compatible with serum. The interactions of the cationic polymer and serum may thus be used as a predictive model for *in vivo* transfection efficiency of the polymer [30]. BPEA exhibited a high transfection efficiency even in the presence of 10% FBS (Fig. 7). In B16-F0 cells, the transfection efficiency obtained by BPEA in the presence of serum was approximately 3-fold higher than that obtained by Fugene HD and PEI 25 kDa, and 400-fold higher than that achieved by naked DNA. Such a high transfection efficiency of BPEA was also observed in HepG2 and U87, in which the transfection efficiency of the polymer was comparable to, or even higher than, that achieved by Fugene HD and PEI 25 kDa, and was further confirmed by the luciferase activity assay (Fig. 8). This result shows that BPEA is a polymer showing high efficiency in gene delivery to a broad range of cell types, and the polymer has promising applications for the future development of gene therapies for different cancers.

## Conclusions

BPEA is a cationic polymer synthesized for gene delivery. BPEA has high pH buffering capacities and DNA condensation abilities, and has low cytotoxicity over a wide range of working concentrations. In addition, this polymer allows efficient transfection of various cancer cell lines, and this high transfection efficiency persists in the presence of serum. In order to further augment the performance, the detailed mechanisms of interaction between plasmids and BPEA, as well as changes in structural parameters of BPEA after complexation with DNA, are worth elucidation in future research. This provides insights into proper design and engineering of the polymeric vector. In addition, the extent of cross-linking of PEI during synthesis of BPEA is worth further optimization. The importance of this has been revealed by earlier studies, which suggested that while cross-linking of PEI on one hand could enhance the transfection efficiency [31], [32], it could also alter the pK profile of the PEI amines, reduce the efficiency of endosomal release and ultimately have a negative impact on transfection [33]. With further improvement and optimization, it is envisaged that BPEA will emerge as an effective gene carrier for *in vitro* and *in vivo* applications, as well as for future cancer gene therapy.
